# Comment on “DeepSeek-assisted LI-RADS classification: AI-driven precision in hepatocellular carcinoma diagnosis

**DOI:** 10.1097/JS9.0000000000003579

**Published:** 2025-10-06

**Authors:** Minghao Shen, Huafeng Gu, Jianhua Xu

**Affiliations:** a Department of Infectious Diseases, Yangming Hospital Affiliated to Ningbo University, Ningbo City, Zhejiang Province, China; b Radiology Department, Yangming Hospital Affiliated to Ningbo University, Ningbo City, Zhejiang Province, China

*Dear Editor,*

We read with great interest the recent study evaluating the application of the DeepSeek-V3 (DSV3) model in LI-RADS categorization for patients at high risk of hepatocellular carcinoma^[1]^. The authors demonstrated that DSV3 outperformed junior radiologists in LR-3 to LR-5 categories while achieving diagnostic accuracy comparable to senior radiologists. We commend the rigorous methodology and insightful analysis presented. While this represents an important advance, several considerations warrant further discussion.

The primary comparison emphasized DSV3’s superiority over junior radiologists. Although this highlights its potential for training purposes, the broader clinical value may lie in whether the model can complement or refine expert judgment, particularly for indeterminate lesions within LR-2 to LR-4. Evaluating its ability to reduce interobserver variability among experienced radiologists would provide stronger evidence for clinical integration.

The study reported comparable accuracy in Chinese and English reports. However, reporting styles in clinical practice vary widely, ranging from structured templates to brief descriptive notes. Structured reports provide standardized inputs for AI, which enhances model stability and reproducibility, whereas free-text narratives pose challenges for Natural Language Processing (NLP) processing and introduce risks of performance variability^[2,3]^. Testing DSV3 across these heterogeneous contexts is essential to determine whether its robustness extends across institutions and healthcare systems, rather than being limited to a specific reporting culture.

Beyond accuracy, the adoption of AI tools depends on transparency and usability. It is important to clarify whether DSV3 can provide explanations for its classifications, how it resolves discrepancies with human readers, and whether it can be seamlessly integrated into PACS or structured reporting systems. Increasingly, studies underscore the necessity of “explainable AI” in medical imaging to mitigate the risk that opaque outputs undermine clinical trust^[4,5]^. These aspects will be critical in determining whether the model can serve as a reliable partner in decision-making.

While the single-center design and modest sample size support proof of concept, they limit external validity. Multicenter validation is indispensable, particularly with cohorts involving small or atypical lesions that often create diagnostic uncertainty. Stratified analyses by imaging modality, lesion size, and underlying liver disease could also clarify the clinical scenarios where DSV3 provides the greatest benefit.

In conclusion, this study highlights the promise of large language model-based tools in LI-RADS classification. To firmly establish clinical value, future research should explore mechanisms for consensus-building among senior radiologists, evaluate robustness across reporting systems and languages, and address challenges of interpretability and workflow integration. Multicenter validation in complex patient populations will be key to ensuring that models like DSV3 advance beyond technical accuracy to deliver meaningful improvements in patient care. This manuscript has been prepared to meet journal requirements. In accordance with the TITAN Guidelines^[6]^, the TITAN checklist has been submitted.

## Data Availability

Not applicable.
